# High Phenobarbital Clearance During Continuous Renal Replacement Therapy: A Case Report and Pharmacokinetic Analysis

**DOI:** 10.1097/MD.0000000000000046

**Published:** 2014-07-25

**Authors:** Staffan Rosenborg, Lars Saraste, Katarina Wide

**Affiliations:** Departments of Clinical Pharmacology (SR) and Anesthesia and Intensive Care (LS), and Neuropediatrics (KW), Karolinska University Hospital, Huddinge, SE-141 86 Stockholm, Sweden.

## Abstract

Phenobarbital is an old antiepileptic drug used in severe epilepsy. Despite this, little is written about the need for dose adjustments in renal replacement therapy. Most sources recommend a moderately increased dose guided by therapeutic drug monitoring.

A 14 year old boy with nonketotic hyperglycinemia, a rare inborn error of metabolism, characterized by high levels of glycine, epilepsy, spasticity, and cognitive impairment, was admitted to the emergency department with respiratory failure after a few days of fever and cough. The boy was unconscious at admittance and had acute renal and hepatic failure.

Due to the acute respiratory infection, hypoxic hepatic and renal failure occurred and the patient had a status epilepticus.

The patient was intubated and mechanically ventilated. Continuous renal replacement therapy was initiated. Despite increased phenobarbital doses, therapeutic levels were not reached until the dose was increased to 500 mg twice daily. Therapeutic drug monitoring was performed in plasma and dialysate. Calculations revealed that phenobarbital was almost freely dialyzed.

Correct dosing of drugs in patients on renal replacement therapy may need a multidisciplinary approach and guidance by therapeutic drug monitoring.

## BACKGROUND

Phenobarbital is an old anticonvulsant still in widespread clinical use.^1^ It is readily absorbed and mainly eliminated by hepatic metabolism, but about 25% of an oral dose is excreted unchanged in urine.^2^ Its molecular weight is 232.24 g/mol.^3^ Figures of protein binding vary from 40–60%.^4–8^ It has been shown that continuous veno-venous hemofiltration may be an effective means of elimination of phenobarbital in cases of intoxication.^9^ Recommendations for dose adjustment in renal replacement therapy vary among different sources from slightly reduced to 5 times increased dose.^4–7^

## CASE DESCRIPTION

The patient was a 14-year-old boy (weight 41 kg, height 140 cm) with nonketotic hyperglycinemia (NKH), a rare autosomal recessive inborn error of metabolism, which leads to neurological deficits and severe epilepsy. The disease can present itself either in the newborn infant or as late onset NKH. All patients show signs of hypotonia, which later evolves into spasticity. The cognitive development is delayed. The primary lesion is a defect in the glycine cleavage system, a mitochondrial enzyme complex, which leads to increased concentrations of glycine in plasma, urine, and cerebrospinal fluid. The pathophysiology remains obscure; the increased levels of glycine may increase the *N*-methyl-d-aspartate (NMDA) receptor activation and lead to an increased concentration of glutamate. There is no treatment apart from low protein diet, to keep the levels of glycine low. NMDA receptor antagonists such as dextromethorphan have been tried. The epilepsy is treated with antiepileptic drugs, and ketogenic diet has also been tried.^10^

The patient was treated with ketogenic diet and several anticonvulsants (clobazam, lorazepam, levetiracetam, and felbamate) including an oral daily dose of 135 mg phenobarbital. In February 2013, he was admitted to the intensive care unit due to a severe infection with respiratory syncytial virus. He had acute hepatic and renal dysfunction, probably due to respiratory failure. His renal function deteriorated and he was started on continuous veno-venous hemodialysis the day after admission. Blood flow was 60 mL/min, dialysate flow (*Q*_D_) 1400 mL/h. Regional citrate anticoagulation was used with an infusion rate of 119 mL/h of sodium citrate 0.13 mmol/L and 11 mL/h of calcium glubionate 9 mg/mL. Net ultrafiltration rate (UFR) was 50 mL/h. Total convective flow can thus be calculated to be 50 + 119 + 11 mL/h = 180 mL/h.

Despite increasing doses of intravenously administered phenobarbital, concentrations fell from 146 μmol/L on oral phenobarbital to 24 μmol/L. After several dose adjustments he was stabilized at a concentration around 100–120 μmol/L on an intravenous dose of 900–1000 mg/day.

## PHARMACOKINETIC ANALYSIS

Systemic clearance of phenobarbital at baseline can be calculated from the relationship *C*_ss_* *= (*F* × Dose)/(*Cl* × *τ*), where *C*_ss_ is steady-state concentration, *F* is bioavailability, and *τ* is the dose interval.

By rearrangement, clearance (*Cl*) can be calculated to be 
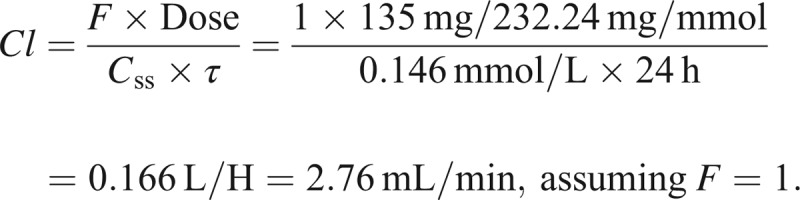


As renal clearance normally is about 25% of systemic clearance, we did not beforehand expect dramatic changes in phenobarbital pharmacokinetics during renal replacement therapy.

**Figure 1:**
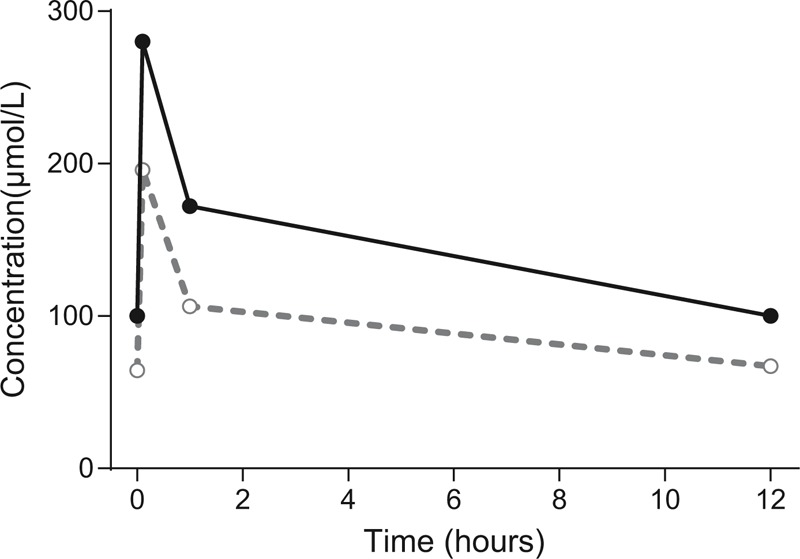
A dose of 500 mg phenobarbital was given at time 0. Samples from plasma and dialysate were drawn predose, at 2 minutes, 1 hour, and 12 hours postdose. Plasma concentrations are shown in black filled circles (●) and dialysate concentrations are shown in grey open circles (○).

Samples were drawn from plasma and dialysate 12 hours after the preceding dose, and 2 minutes, 1 hour, and 12 hours after a new intravenous dose of 500 mg (Figure 1). Therapeutic drug monitoring was performed with the method currently in use at the Karolinska University Laboratory (Cloned enzyme donor immunoassay Phenobarbital II Assay, Microgenics Corporation, Fremont, CA). Dialysate samples were analysed undiluted and diluted 1:2 and 1:4 in blank plasma in order to estimate potential matrix effects. Measurements of the different dilutions agreed within a coefficient of variation of 7.5%.

Areas under the concentration–time curves (AUC_0–12 h_) were calculated according to the trapezoidal rule to be 1718 μmol × h/L in plasma and 1103 μmol × h/L in dialysate.

Total plasma clearance could be calculated to be dose/AUC, that is, 0.5 × 232.2/1718 = 1.27 L/h = approximately 21 mL/min.

Total extracorporeal clearance (*Cl*_EC_) could be calculated according to the formula 
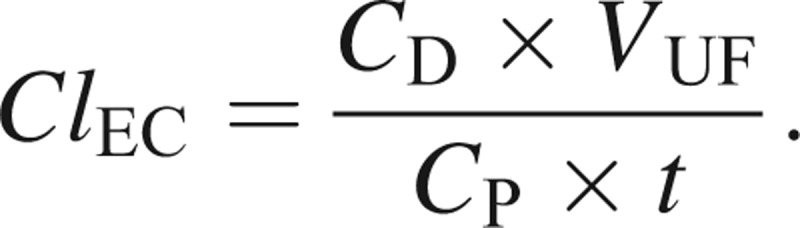


Since *C*_P_* × t = *AUC_P_, the mean dialysate concentration is given by *C*_D_* = *AUC_D_/*t*, and *V*_UF_* = *(*Q*_D_* + *UFR)* × t*, 
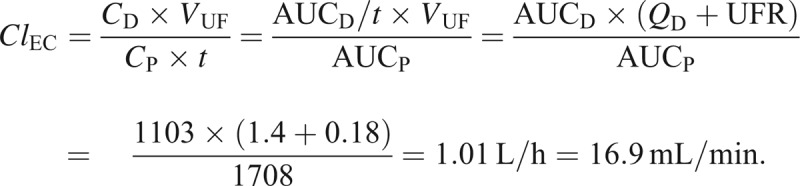


## DISCUSSION

Phenobarbital clearance was dramatically increased during continuous veno-venous hemodialysis. Extracorporeal clearance alone was 6-fold higher than baseline systemic clearance and systemic clearance during renal replacement therapy was 7.5-fold higher than at baseline. During complete equilibration between blood and dialysate, theoretical dialysis clearance can be calculated as the product between dialysate flow and unbound fraction, that is, 1400 mL/h × (0.4 − 0.6) = 9.3 − 14 mL/min. Theoretical convective clearance can similarly be calculated as the product of sieving coefficient and UFR. In the case of phenobarbital, the sieving coefficient has been measured to be about 0.8,^7^ and the total UFR would be the sum of the infusion rates of calcium and citrate and the net ultrafiltration. Theoretical convective clearance would, therefore, be 144 mL/h = 2.4 mL/min. Total theoretical clearance would then be 11.7–16.4 mL/min. This is very close to the observed clearance in our case.

This case illustrates that although a drug has a relatively low renal clearance, its clearance by continuous dialysis may be considerably higher. The difference may be explained by tubular reabsorption that decreases renal clearance and that may be amplified in ketogenic conditions.

Sources for dose recommendations in renal disease differ considerably regarding their recommendation in continuous renal replacement therapy (CRRT). One source even states that phenobarbital is not dialyzed by CRRT.^5^ Other sources recommends supplementation doses after hemodialysis,^6,8^ and a fourth differs considerably between adult and pediatric patients in recommended dosing during CRRT,^4^ where children are recommended an up to 5-fold higher weight-adjusted dose in CRRT, whereas adults are not.

This case description illustrates that it may be difficult to decide a priori the pharmacokinetic consequences of initiating renal replacement therapy. Proper dose adjustments will depend on an adequate estimation of the individual patient and the intended parameters of the renal replacement therapy. We would like to stress the need for therapeutic drug monitoring and a multidisciplinary approach to patient care.
